# Editorial

**Published:** 1901-10-15

**Authors:** 


### EDITORIAL.

Considerable interest was manifested in the practically unanimous vote by which the members of the National Faculties Association, at its recent annual meeting, decided to extend the course of study to four terms of seven months each. The Southern schools wanted a six months term, because of the difficulties of carrying on the work during the warm spring months; but they reluctantly waived this objection, and voted with the majority. The sentiment was practically unanimous that it was a step in the right direction and wisely taken. By this decision the four term course is set for the session beginning the fall of 1903. In this connection it may be interesting to notice the result of the inauguration of the four year course at the University of Michigan. At this school not only a four year course has just gone into effect, but the entrance requirements have also been raised to a high school graduation. The effect upon the entering or freshman class has been to materially reduce it in numbers. For the past two years this class numbered, respectively, ninety and ninety-five; but this year it has dropped to thirty-five. Should a similar reduction seem probable in other schools when they begin their four year courses, it is highly probable there will be an effort made to rescind the resolution passed this year. Fortunately such a result may not be expected when the four term becomes established in all schools.

The University of Michigan and the Detroit College of Medicine only, of the fifty dental schools in the country, have taken this advance, and it was to be expected that a heavy falling off in attendance should occur. It would have been more desirable had a larger number of schools taken the step at this time, but every effort that could be made was employed to induce others to do so, particularly the stronger schools. Failing to secure this co-operation, the University of Michigan decided after mature deliberation to undertake the step alone. While the number matriculating for the four year course would seem to indicate, at least from a commercial point of view, that the step was hasty and unwise, the high standard of preparation secured, together with the earnest spirit shown by the class, leaves little question but that time will justify the wisdom of the movement. It places a grave responsibility upon the teaching faculty which assumes to administer a curriculum broad enough to meet the demands of the times. It must also mean a real advance, in order that students shall receive adequate compensation for the time and expense involved.

---

The Secretary of the National Association of Dental Examiners requests us to invite the executive officers of the various State Boards of Examiners to send the names of their members and officers to Dr. J. A. Osmun, 588 Broad Street, Newark, N. J. The possession of this information will enable him to keep in touch with all interested in this important work, and also to secure such information as shall enable him to administer his executive duties in an effective manner.

